# A Review of the Important Role of *CYP2D6* in Pharmacogenomics

**DOI:** 10.3390/genes11111295

**Published:** 2020-10-30

**Authors:** Christopher Taylor, Ian Crosby, Vincent Yip, Peter Maguire, Munir Pirmohamed, Richard M. Turner

**Affiliations:** 1 Wolfson Centre for Personalised Medicine, University of Liverpool, Liverpool L69 3BX, UK; vincent.yip@liverpool.ac.uk (V.Y.); munirp@liverpool.ac.uk (M.P.); rt34@liverpool.ac.uk (R.M.T.); 2 MC Diagnostics, St Asaph Business Park, Saint Asaph LL17 0LJ, UK; ian.crosby@mcdiagnostics.co.uk (I.C.); peter.maguire@mcdiagnostics.co.uk (P.M.)

**Keywords:** *CYP2D6*, pharmacogenomics, drug metabolism, population variability, structural variation

## Abstract

Cytochrome P450 2D6 (*CYP2D6*) is a critical pharmacogene involved in the metabolism of ~20% of commonly used drugs across a broad spectrum of medical disciplines including psychiatry, pain management, oncology and cardiology. Nevertheless, *CYP2D6* is highly polymorphic with single-nucleotide polymorphisms, small insertions/deletions and larger structural variants including multiplications, deletions, tandem arrangements, and hybridisations with non-functional *CYP2D7* pseudogenes. The frequency of these variants differs across populations, and they significantly influence the drug-metabolising enzymatic function of CYP2D6. Importantly, altered CYP2D6 function has been associated with both adverse drug reactions and reduced drug efficacy, and there is growing recognition of the clinical and economic burdens associated with suboptimal drug utilisation. To date, pharmacogenomic clinical guidelines for at least 48 CYP2D6-substrate drugs have been developed by prominent pharmacogenomics societies, which contain therapeutic recommendations based on CYP2D6-predicted categories of metaboliser phenotype. Novel algorithms to interpret CYP2D6 function from sequencing data that consider structural variants, and machine learning approaches to characterise the functional impact of novel variants, are being developed. However, *CYP2D6* genotyping is yet to be implemented broadly into clinical practice, and so further effort and initiatives are required to overcome the implementation challenges and deliver the potential benefits to the bedside.

## 1. Introduction

### 1.1. Background to Cytochrome P450 Enzymes

The cytochrome P450 (CYP) superfamily is an ancient family of enzymes identified in hundreds of eukaryote and prokaryote species [1,2], and are named thus because they strongly absorb 450 nm wavelength light when reduced and bound by carbon monoxide. CYP standard nomenclature is the prefix, “CYP”, followed by a number for the family (proteins sharing more than 40% amino acid sequence identity), a letter for the subfamily (at least 55% identity) and a number for the specific gene; for example, *CYP2D6* [3]. The human genome encodes 57 putatively functional *CYP* genes along with 58 pseudogenes [4]. Of these 57 functional human CYPs, 12 are implicated in the metabolism of 70–80% of commonly used drugs; specifically, these are: CYP1A1, CYP1A2, CYP1B1, CYP2A6, CYP2B6, CYP2C8, CYP2C9, CYP2C19, CYP2D6, CYP2J2, CYP3A4 and CYP3A5 [4].

CYPs are hemoproteins and there is extensive diversity of CYP gene sequences [5,6]. However, the three-dimensional (3D) structure of the common CYP fold is highly conserved, being predominantly α-helical (usually 12–13 α-helices) with a small number of β-sheets [5,6,7]. The structural domains that surround the heme complex form the substrate recognition and access channel [3]. CYP enzymes are typically the terminal oxidase enzymes in electron transfer chains. The major electron donor for microsomal CYPs is P450 reductase (POR), which donates the first and often both of the electrons required by a CYP for catalysis [8,9]. The canonical reaction catalysed by CYPs is monooxygenation, although they can also catalyse other reactions including hydroxylation and dealkylation. Human and other mammalian CYP enzymes are membrane-anchored proteins embedded in the phospholipid bilayers of the endoplasmic reticulum or mitochondria [5,10].

### 1.2. Background to Pharmacogenomics

Interindividual variability in drug response is a growing concern for healthcare systems across the world, since it can blunt drug efficacy and precipitate adverse drug reactions (ADRs) leading to patient harm, poorer health outcomes, and inefficient consumption of limited healthcare resources. It is estimated that for many drugs used across a range of diseases, only 50–75% of patients experience a beneficial response [11]. Studies in European populations have demonstrated that 3.5–6.5% of hospitalised patients are admitted because of ADRs, and one or more ADRs can occur in almost 15% of hospital inpatients [12,13,14]. Additionally, the risk of ADRs increases in patients administered multiple drugs [15], contributing to the clinical burden of (potentially inappropriate) polypharmacy. ADRs differ in severity with the majority of adverse events considered mild (e.g., headaches, fatigue, constipation and/or nausea), although the overall number of cases is believed to be underreported [16]. Nevertheless, non-severe and non-serious cases still present an avoidable burden to healthcare systems. Notably, a 2015 National Institute for Health and Care Excellence (NICE) statement concluded that 72% of ADRs are potentially avoidable, with the estimated cost of these ADRs in excess of £500 million in the UK alone [17]. Therefore, it is important to understand and mitigate drug response variation.

Variation in drug response is, in general, multifactorial with demographic, environmental, clinical, and genomic components. Pharmacogenomics is the study and clinical application of the genetic determinants of drug response. The aim of pharmacogenomics is to guide dose selection, drug selection, and/or prioritise patients for closer monitoring through stratification of patients based on genetic variants in order to improve drug efficacy and/or safety, and this is at the vanguard of the precision medicine initiative [18,19,20]. Pharmacogenomic studies have evolved from candidate gene investigations to genome-wide association studies (GWAS) and increasingly to sequencing based projects; sample sizes have similarly grown although, in general, remain notably smaller compared to genomic studies of polygenic diseases. Nevertheless, the magnitude of drug–gene associations is often notably larger than disease–gene associations [21], presumably reflecting less constrained evolutionary selection pressures. Currently, pharmacogenomic information is contained somewhere within the drug label of 298 drugs approved by the US Food & Drug Administration (FDA) [22]. Moreover, to date, the clinical application of gene associations with 123 drugs has been reviewed and, of these, clinical guidelines for just over 80 drugs have been developed that contain clinically actionable pharmacogenomic recommendations [23]. The majority of this evidence assessment and guideline writing has been conducted by the Royal Dutch Association for the Advancement of Pharmacy-Pharmacogenetics Working Group (DPWG) [24] and the Clinical Pharmacogenetics Implementation Consortium (CPIC) [25], although other guideline-writing committees also exist such as the Canadian Pharmacogenomics Network for Drug Safety (CPNDS) [26], the Réseau National de Pharmacogénétique (RNPGx) [27], and the NHS England Pharmacogenomics working group.

### 1.3. Background to CYP2D6

CYP2D6 is a particularly important and well-studied member of the CYP superfamily. In the late 1970s, the metabolism of debrisoquine, and separately sparteine, were both shown to be highly variable yet controlled by a single autosomal gene, which we now know to by *CYP2D6* [28,29]. The human *CYP2D6* gene is relatively short, spanning just ~4.3 Kbps on the long arm of chromosome 22 (22q13.2), is encoded by nine exons, is translated into CYP2D6 protein that localises to the endoplasmic reticulum, and is highly expressed in liver, brain, intestinal tissue and lymphoid cells [10,30]. *CYP2D6* has numerous endogenous substrates including tyramine in the brain and lymphocytes [31,32], and 5-hydroxyindoleacetic acid in brain tissue and possibly cerebrospinal fluid [33,34]. Importantly, although CYP2D6 constitutes just 2–4% of total hepatic CYP content [35], it is a cardinal drug-metabolising enzyme involved in the metabolism of approximately 20% of commonly used drugs [36,37]. Thus, CYP2D6 can metabolise a wide range of substrates including analgesics (e.g., codeine, tramadol), antidepressants (e.g., paroxetine, tricyclic antidepressants), antihypertensives (e.g., metoprolol, bisoprolol) and the anti-cancer agent, tamoxifen [38,39,40,41].

**Figure 1:**
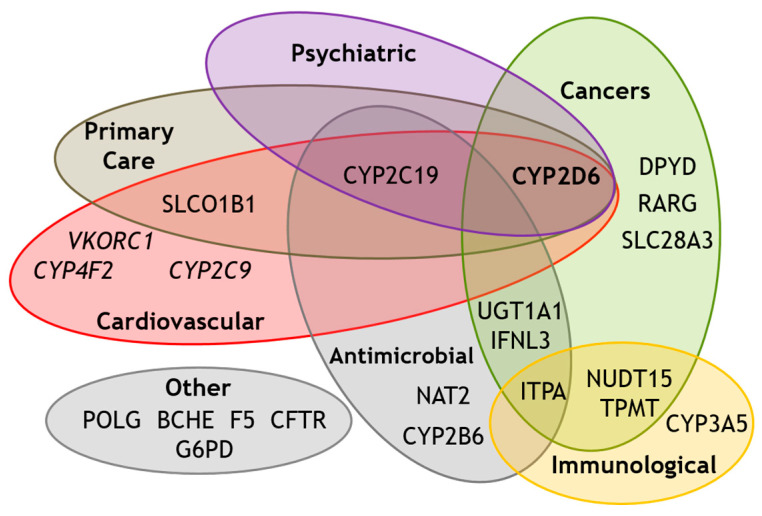
Venn Diagram showing recognised (non-*HLA*) pharmacogenes according to their association with drugs used in different therapeutic areas. *CYP2D6* overlaps with four distinct therapeutic areas. *BCHE*: Butyrylcholinesterase. *CFTR*: Cystic fibrosis transmembrane conductance regulator. *CYP2B6*, *CYP2C9*, *CYP2C19*, *CYP3A5*, *CYP4F2*: Members of cytochrome P450 superfamily. *DPYD*: Dihydropyrimidine dehydrogenase. *F5*: Factor 5. *G6PD*: Glucose-6-phosphate dehydrogenase. *IFNL3*: Interferon lambda 3. *ITPA*: Inosine triphosphate pyrophosphohydrolase. *NAT2*: N-Acetyltransferase 2. *NUDT15*: Nudix hydrolase 15. *POLG*: DNA polymerase subunit γ. *RARG*: Retinoic acid receptor γ. SLCO1B1: Solute carrier organic anion transporter family member 1B1. *SLC28A3*: Solute carrier family 28 member 3. TPMT: Thiopurine S-methyltransferase. *UGT1A1*: UDP-glucuronosyltransferases. *VKORC1*: Vitamin K epoxide reductase complex.

Overall, to date, 72 different drugs have *CYP2D6* mentioned within their FDA-approved product label [42]. Furthermore, the clinical relevance of *CYP2D6* to at least 48 drugs has been reviewed by pharmacogenomic guideline committees and actionable recommendations based on *CYP2D6* have been developed for 26 drugs to date [23], as summarised in Table 1. It is clear that *CYP2D6* is a promiscuous pharmacogene implicated in drug response across a large number of medical specialities (Figure 1).

The rate of CYP2D6-mediated microsomal metabolism varies at least 60-fold between individuals [43]. *CYP2D6* is highly polymorphic and its genetic complexity is an important contributor to its functional variation. Therefore, the genetic complexity of *CYP2D6* and its pivotal role in the metabolism of multiple drugs makes accurate and effective *CYP2D6* genotype-based clinical prescribing a key milestone in any pharmacogenomics implementation effort. Thus, this review will focus on describing *CYP2D6* genomic variation, the functional consequences of this variation, laboratory methods to detect and analyse *CYP2D6* variants, the clinical impact of *CYP2D6* pharmacogenomics, and highlight cutting edge developments in parsing *CYP2D6* genotype-to-phenotype translation. This review has been written for an intended audience that includes both non-geneticists and healthcare prescribers as well as those proficient in pharmacogenomics.

## 2. An Overview of *CYP2D6* Variation

Variation in *CYP2D6* includes single-nucleotide variants (SNVs), indels, whole-gene deletions, multiplications, tandem arrangements and hybridisations [40,44]; it can also be affected by larger chromosomal deletions or other structural alterations as part of the rare 22q13 deletion syndrome, known as Phelan-McDermid syndrome. Currently, there are 133 *CYP2D6* “star” (“*”) alleles listed on the PharmVar data repository [45], as detailed within a recent and in-depth review of 2D6 nomenclature and 2D6 allele designation by Nofziger et al. [46]. In essence, the star allele nomenclature system represents a useful haplotype-based system that is well established and recognised within the pharmacogenomic field [46]. Many of the listed variants contribute in part or entirely to altered rates of CYP2D6-associated drug metabolism. The majority of identified *CYP2D6* variants are SNVs, which encompass both single-nucleotide polymorphisms (SNPs, defined as SNVs with a minor allele frequency, MAF ≥ 1%) and rarer (MAF < 1%) nucleotide substitutions. Like other genes, *CYP2D6* SNVs can be in the non-coding regions (e.g., the promoter or introns) with variable effects on protein expression, or located in the coding regions, leading to synonymous or nonsynonymous (both missense and nonsense) alterations. Small base insertions/deletions (indels) may be of little consequence when occurring in non-coding (e.g., intronic) gene regions, although they could perturb transcription factor binding. However, when indels affect coding DNA and are not a multiple of three, they can change the reading frame and result in frameshift mutations. Importantly, beyond these short forms of variation, the genetic architecture of *CYP2D6* differs from that of other *CYP* pharmacogenes by the extent and complexity of larger structural variation that can occur, as briefly highlighted below. For further information, the reader is directed to the Pharmacogene Variation Consortium (PharmVar) *CYP2D6* Structural Variation document [47], which provides an excellent up-to-date and detailed overview of *CYP2D6* structural variation.

### 2.1. Pseudogenes

**Figure 2:**
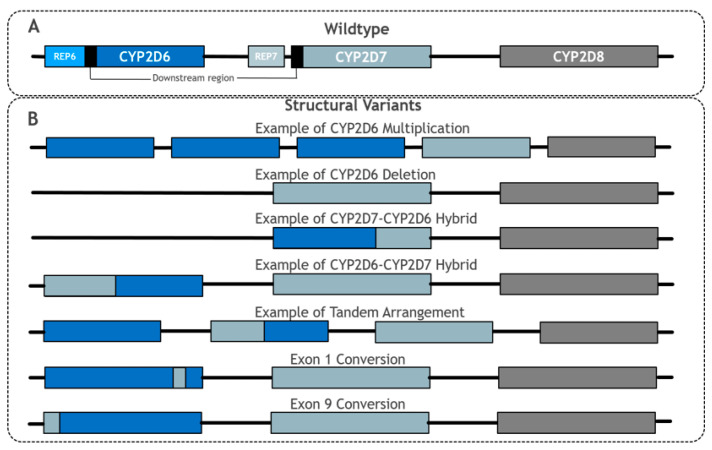
Summary of main CYP2D6 structural variant types. Adapted from PharmVar CYP2D6 Structural Variation document [47]. (**A**) shows the wildtype layout of CYP2D6, CYP2D7 and CYP2D8. (**B**) shows examples of structural variants involving CYP2D6. For simplicity, REP6, REP7 and downstream elements have been omitted in (**B**).

One major complication of analysing *CYP2D6* is resolving the issue of the highly similar non-functional pseudogenes, *CYP2D7* and *CYP2D8*, which are located nine and 19 kbp upstream of *CYP2D6* and have 94.2% and 89.1% sequence similarity to *CYP2D6*, respectively [36]. All three genes consist of nine exons, and *CYP2D6* and *CYP2D7* share a common downstream element that is 0.6 kbp in length [43,47]. Moreover, *CYP2D6* and *CYP2D7* have near-identical repetitive sequences termed REP6 and REP7, respectively, although REP7 is separated by an additional short length of sequence from the common element in *CYP2D7*, unlike REP6 in *CYP2D6* [47] (see Figure 2A). This level of similarity, particularly between *CYP2D7* and *CYP2D6*, requires any genotype detection method to be highly specific to *CYP2D6* [36]. However, further complicating this problem is that many haplotype-defining *CYP2D6* SNPs, such as rs35742686 (*3), have identical flanking sequences in *CYP2D6* and *CYP2D7*, and thus present a challenge to distinguish between using probe-based and microarray testing techniques.

### 2.2. Copy Number Variation

Copy number variation (CNV) is a type of larger structural variation and, in a diploid genome, generally refers to the presence of more or less than two copies of a gene. CNV is common in *CYP2D6* and individuals, with one or three *CYP2D6* copies occurs at a frequency of 12–23% depending on population ethnicity [40]. The significance of this type of variation on the metaboliser phenotype of CYP2D6 depends on the functionality of the duplicated *CYP2D6* allele. Indeed, it has been shown in a population of patients from Hong Kong that fewer than 20% of duplicated *CYP2D6* alleles were duplications of functional alleles [44]. However, an increased copy number of functional *CYP2D6* alleles increases the rate of metabolism of associated drugs [39], with some studies reporting as many as 13 copies of *CYP2D6* in a single individual’s genome [48]. Thus, an individual with more than two functional copies of *CYP2D6* is considered to have ultra-rapid metabolism for CYP2D6 substrate drugs. CYP2D6*1, *2 and *4 are the most common haplotypes in which duplications or multiplications are observed and are denoted as ‘CYP2D6*1xN’ ‘*2xN’ and ‘*4xN’, respectively [40]. Other rarer duplications have been identified, including CYP2D6*6xN, *10xN, *17xN, *36xN, *41xN, *43xN and *45xN [49].

CYP2D6*5 represents a whole allele deletion with a corresponding complete loss of function of the allele. Overall, this variant is present in 3–6% of African, European and East Asian populations (Table 2). However, it is more diverse within sub-populations and so, for example, gradually increases from 1 to 6%, moving from southern to northern European countries, respectively [50]. CYP2D6*5 deletions are derived from breakpoints in the highly repetitive REP6 and REP7 sequences [38], leaving CYP2D6*5 with a REPdel sequence consisting of fused REP6 and REP7 elements [51].

### 2.3. Hybridisations, Tandem Arrangements and Conversions

Structural hybridisation describes the rearrangement of a gene sequence to form an alternate gene sequence, which often leads to a protein with reduced or no function. Many different hybridisations of *CYP2D6* have been observed and typically involve the joining of *CYP2D6* and *CYP2D7* sequences together to make a recombinant hybrid gene (Figure 2B) [51,52,53,54]. *CYP2D6-2D7* and *CYP2D7-2D6* are two distinct forms of hybridisation (see below). It is also possible that a sequence from *CYP2D7* can be inserted into *CYP2D6* at a specific point, which is termed a gene conversion [36,47].

*CYP2D6-2D7* hybrids are formed when the 5′ section is from *CYP2D6* and the 3′ section is from *CYP2D7*, forming a hybrid structure in place of wildtype *CYP2D6*. Examples of this include *36 and *61. Similarly, in *CYP2D7-2D6* hybrid genes, the 5′ element of the hybrid is from *CYP2D7* and the 3′ part is from *CYP2D6*; *CYP2D7-2D6* arrangements lack *CYP2D7* 5′ to the recombinant gene and so could originate from the deletion of the intervening sequence [53]. Several *CYP2D7-2D6* hybrids that conform to this pattern have been identified; although they differ in the exact *2D7-2D6* joining point between the two genes, they are collectively grouped together under the CYP2D6*13 designation [47].

Tandem arrangements contain two or more copies of a gene unit on the same chromosome featuring non-identical variation between the gene units. Gene duplications and multiplications are distinguished from tandems by the gene units of duplications/multiplications being the same [47]. In most tandems, at least one gene copy is a *CYP2D7-2D6* or *CYP2D6-2D7* hybrid arrangement. CYP2D6*36 + *10 is the most common tandem arrangement, and is particularly prominent in East Asian populations with an MAF of 34.1% [44]. In this tandem arrangement, the 5′ upstream gene unit is usually a *2D6-2D7* hybrid (*36), whilst the 3′ downstream gene unit contains the missense SNP that defines *10 (rs1065852, p.P34S). Evidence suggests that the *36 + *10 tandem is associated with decreased function, reflecting that *36 alone (as a single gene unit) has no function and *10 alone has decreased function [36,55,56].

### 2.4. Structural Impact of Star Alleles

**Figure 3:**
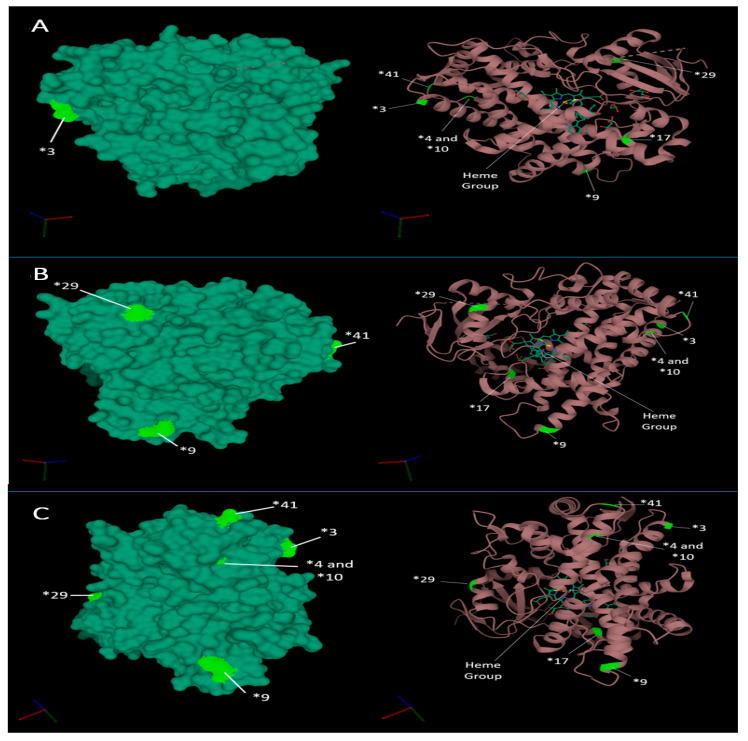
The 3D structure of CYP2D6 from different angles, annotated with the locations of notable star allele-defining variants (neon green). Images (**A**–**C**) show CYP2D6 from different angles. The images on the left and right show the molecular surface of CYP2D6 and ribbon model of CYP2D6, respectively. The central heme group is highlighted in the ribbon models. Molecular structures were generated and annotated using Research Collaboratory for Structural Bioinformatics (*RCSB*) Protein Data Bank (PDB) software [59].

X-ray crystallography has revealed that the 3D structural conformation of CYP2D6 differs between ligand-bound and ligand-free forms. The most distinct structural changes observed include the closing of the active site by reorganisation of helix F and Gly-218 [57,58]. The structural changes caused by the non-synonymous, frameshifts and splicing mutations, which define the CYP2D6 star allele, drive the change in function, thus driving the metabolism phenotype. However, several of the most common function effecting point mutations do not occur near amino acids associated near the active site, instead occurring towards the extremities of the molecule, distant form the central heme iron (Figure 3).

## 3. CYP2D6 Metaboliser Status

The current categorisation of CYP2D6 metaboliser status is based on activity scoring of known haplotypes [46]. Activity scores (AS) serve as a useful tool to translate information regarding the function of individual haplotypes into an overall predicted metaboliser status for a given diplotype, and thus an individual. At present, *CYP2D6* categories remain relatively discrete and are broadly grouped into poor metaboliser (PM, AS = 0), intermediate metaboliser (IM, AS = 0.25–1), extensive (normal) metaboliser (EM, AS = 1.25–2.25) and ultra-rapid metaboliser (UM, AS > 2.25) strata [60]. This pragmatic approach is useful and is periodically updated as evidence regarding the metaboliser function of individual haplotypes accumulates. *CYP2D6* pharmacogenomic guidelines use these metaboliser categories to provide clinicians with drug-specific recommendations with the aim of avoiding doses or drugs expected to be ineffective or harmful in patients that have a particular *CYP2D6* metaboliser status.

## 4. *CYP2D6* Star Allele Frequencies

Table 2 shows population frequencies for several *CYP2D6* star alleles (that generally represent haplotypes) that are common (MAF ≥ 1%) in at least one major population group and (except *2) affect CYP2D6 function. However, importantly, these crude frequencies do not account for the complexities of *CYP2D6* haplotype impact on metabolism. For example, rare variants are not considered. Furthermore, in-depth studies assessing overall frequencies of CYP2D6 metabolism status found that >20% of Ashkenazi Jewish and Europeans populations have non-functional haplotypes, 45% of East Asian Individuals had reduced function haplotypes and >10% Oceania individuals have increased function haplotypes [61]. This high haplotype frequency disparity can also be seen in other sub-populations and some haplotypes are only present at high frequency in specific populations. For example, CYP2D6*4 was found to be present in 22% of Jewish individuals [61] and CYP2D6*5 was found to be present in 16% of north Indian individuals; however, both CYP2D6*4 and CYP2D6*5 were significantly lower in other populations [62]. These population frequencies have also been stratified by AS, which varies depending on the combination of haplotypes and number of duplicates/deletions present. These data showed the high frequency of AS 1.5–2 across most major populations, and AS > 2 in over 10% of Oceanian, Middle Eastern and Ashkenazi Jewish populations [61].

## 5. Detection and Interpretation of *CYP2D6* Genotype

Several existing *CYP2D6* haplotype detection methods exist, including methods which can detect hybrid arrangements and quantify CNVs. Many of these methods include an initial sequencing step to specifically amplify *CYP2D6* prior to genotyping, or as a comparison to other assays. It is important to note that, at present, there is no agreed consensus on which *CYP2D6* variants should be always included when interrogating *CYP2D6*. Therefore, whilst different commercial solutions usually test some major *CYP2D6* alleles (*2, *4, *5, *10, *17), different combinations of other *CYP2D6* variants are provided by different products [65].

Long-range polymerase chain reaction (PCR) or extra-long-range PCR methods are designed to amplify the entire *CYP2D6* gene and therefore can detect multiple copies or whole-gene deletions [66]. Some studies further developed this approach by directly sequencing the PCR product to ensure high sequence fidelity within exonic regions [67]. Pyrosequencing in particular has been established as an inexpensive high-throughput sequencing method in comparison to traditional Sanger sequencing [68] and can been used as a sequencing step for *CYP2D6* variant detection [69]. This sequencing step is then followed by a *CYP2D6*-specific mutant probe detection assay [67]. Combined, these methods are able to accurately determine the presence of a small number of CNVs and has the potential to capture multitudes of other SNPs of interest. However, in its current form, this process is better suited for small-scale research projects, as it consists of multiple PCR steps, and is expensive and labour-intensive.

Taqman methods use bioluminescent tagged probes which can be designed to capture specific sequences with a high degree of precision. qPCR amplification using Taqman probes enables the relative quantification of target sequences, making it ideal for detection of CNVs [70]. However, this method is expensive and labour-intensive if testing a wide variety of haplotypes and has limited multiplexing potential.

Many companies offer custom microarrays which can capture SNPs; however, not all are able to determine CNVs and thus have limited use for clinically meaningful *CYP2D6* genetic analysis. More specialised products are capable of detecting *CYP2D6* CNVs [71]. The Amplichip *CYP2D6* genechip platform is a microarray hybridisation assay that can detect CYP2D6*2, *3, *4, *5, *6, *9, *10, *35 and *41 and duplicates of these SNPs [72]. Similar to Ampligene, *CYP2D6* Genochip is a microarray that can detect CYP2D6*3, *4, *6, *7, *8, *9, *10, *11, *17, *29, *41, gene deletion (*5) and gene multiplication (*xN). Comparisons between these platforms have shown a high degree of concordance; however, the Ampligene platform appears able to elucidate *CYP2D6* diplotypes more precisely [73].

Several algorithms have been developed to infer *CYP2D6* haplotype from whole-genome sequencing data [74,75,76]. Most recently, the Cyrius software algorithm has been shown to be the most accurate with 96.5% concordance between predicted and reported haplotype [76]. This approach could prove to be a valuable way to predict an individual’s metabolism using existing data as whole-genome sequencing becomes more affordable and more commonly undertaken.

Sequencing and genotyping of the somatic genome is becoming increasingly available in oncology. However, there are challenges to using (stored) tumour tissue to determine an individual’s (germline) pharmacogenomic profile. Of note, cancer mutagenesis can potentially alter the genotype of pharmacogenes within the somatic genome, leading to misclassification of the predicted germline genotype. Furthermore, in breast cancer, the region on chromosome 22 that contains *CYP2D6* is commonly deleted [77]. Lastly, the process of formalin fixation damages somatic DNA, although repair processes have been developed [78].

## 6. Factors Impacting CYP2D6 Function beyond *CYP2D6* Genetics

Adding to the complexity of CYP2D6 metabolism is the effect of non-genetic factors that contribute to metaboliser phenotype. As it stands, existing haplotypes alone may not reflect the full underlying genetic complexity of drug-metabolising CYPs due to, for example, rare variation, nor do they consider non-genetic factors. Epigenetic regulation, drug–drug interactions and some foodstuffs influence CYP2D6 activity. Of note, drugs that inhibit CYP2D6 function can lead to an individual having a less functionally active metaboliser phenotype than would be predicted by their genotype, a process termed ‘phenoconversion’ [79,80]. Examples of strong CYP2D6 inhibitors that increase the area under the concentration–time curve (AUC) of sensitive CYP2D6 substrates (e.g., dextromethorphan, nortriptyline, eliglustat) by ≥5-fold include fluoxetine, paroxetine and bupropion [81]. In addition, a range of food products and associated chemicals have also been identified as CYP2D6 inhibitors, including sesamin [82], curcumin and the botanical herb, goldenseal [83]. CYP2D6 phenotyping can evaluate phenoconversion, for example in dedicated drug-drug interaction studies incorporating a CYP2D6 probe drug (e.g., dextromethorphan) or other substrate of interest. Alternatively, endogenous metabolites have been suggested as alternate CYP2D6 probes, such as urinary M1 (*m*/*z* 444.3102) identified through global metabolomics [84], although further validation of endogenous metabolites should be sought. Several proposed methods have been suggested to counteract the issue of phenoconversion by CYP2D6 inhibition including prioritising phenotyping over genotyping where possible, utilising metabolic probes or even using endogenous compounds to determine phenotype. One suggested improvement is simply to evolve the nomenclature to incorporate phenoconversions, therefore separating this from ‘classic’ metabolism statuses (e.g., pUM instead of UM) [85]. This approach would theoretically reduce communication errors and provide additional information when capturing phenoconversion data.

Interestingly, there are no recognised drug inducers of *CYP2D6*, and so the UM phenotype appears predominantly genetic in origin. It appears that canonical xenobiotic-sensing receptors that upregulate expression of *CYP3A4* and other CYPs, including pregnane X receptor (PXR) and constitutive androstane receptor (CAR), have little effect on *CYP2D6* [86]. Nevertheless, the expression of *CYP2D6* transcripts is controlled by different transcriptional regulators and growing evidence suggests they contribute to part of the observed *CYP2D6* variability [43]. For example, the transcription factor, hepatocyte nuclear factor 4α (HNF4α), is a global regulator of genes involved in liver-specific functions, and it has been shown in a systematic study of over 450 human livers that the mRNA levels of HNF4α correlate with the microsomal activity of multiple drug-metabolising CYPs, including CYP2D6 [87]. Moreover, a rich pharmacokinetic study that administered a single dose of tolterodine (a CYP2D6 substrate) to 30 healthy Korean individuals determined that a genetic variant within *HNF4A*, G60D, was significantly associated with tolterodine exposure independent of *CYP2D6* genotype, and accounted for approximately a quarter of the variation explained by *CYP2D6* [88]. It has also been observed that pregnancy induces CYP2D6 activity [43]. Although mechanistic understanding of this is incomplete, mouse models (e.g., CYP2D6-humanised mice) and cell lines (e.g., HepG2 cells) have led to the identification of the novel transcription factors, small heterodimer partner (SHP) and Krüppel-like factor 9 (KLF9), that appear to act as a corepressor and coactivator of HNF4α-mediated transactivation of the *CYP2D6* promoter, respectively [43]. Thus, in pregnancy, it appears that expression of the corepressor SHP is reduced, whilst levels of the coactivator KLF9 are increased, plausibly leading to CYP2D6 induction [89,90].

## 7. CYP2D6 Clinical Impact

### Clinical Guidelines and Pharmacogenomics Implementation

The clinical guidelines developed by both CPIC and DPWG for CYP2D6-substrate drugs have overall a high degree of similarity in their determination of CYP2D6 metabolism status and therapeutic recommendations [91]. Moreover, recent efforts to address inconsistencies between these guidelines have further harmonised CYP2D6 metabolism status [39,60,92]. Nevertheless, the advice between the guideline writing groups differs for a few drugs, including fluvoxamine and paroxetine (Table 1). It is also apparent that few drugs have had guidelines developed for them from all of the aforementioned pharmacogenomic guideline-writing committees, reflecting the international collaborative approach of the relatively small pharmacogenomics research field, the effort and expertise required to develop clinical guidance, and the likely influence of different patterns of prescribing between countries and regions.

As stated earlier, actionable pharmacogenomic guidance has been developed for 26 drugs involving *CYP2D6* to date. The majority of these drugs are established drugs that already have a license. An exception is the glucosylceramide synthase inhibitor, eliglustat, which is a new drug indicated in Gaucher disease type 1, and was licensed with a requirement for companion *CYP2D6* testing, as stated in its product label [93]. The evidence underpinning *CYP2D6* clinical guidelines is generally based on the totality of published, predominantly observational, studies, due to an absence of large randomized control trials. Despite the limitation of this lack of gold-standard evidence, the amalgamation of congruent pharmacokinetic studies, clinical observational studies and case reports within clinical guidance provides a pragmatic approach to sensibly interpret and facilitate the use of the existing knowledge base. It can be contended that this evidence base is equivalent to the evidence underpinning dose/drug modifications based on renal or liver function biochemical testing, which is routinely carried out for many drugs in clinical practice. Overall, the question over the evidential threshold needed for dose/drug optimisation strategies has yet to be fully addressed; however intuitively, it seems reasonable to suggest that, given limited resources for research, it should not always require the same level of evidence as required for tests that carry greater clinical consequences (e.g., tests that lead to a new diagnosis, or, for example, high-risk BRCA mutations that can lead to prophylactic surgery).

Nevertheless, to inform the evidence base for pharmacogenomics implementation, one initiative being conducted is the PREPARE study. PREPARE is a large pan-European block randomised prospective pharmacogenomics intervention study, coordinated by the Ubiquitous Pharmacogenomics consortium, that has recruited ~7000 patients. These patients started one of 42 drugs, for which a DPWG guideline is available, and have been followed up for adverse events for 12 weeks [19]. In the control arm, patients were dosed according to standard care. In the intervention arm, patients have been genotyped for 44 variants in 12 genes, including *CYP2D6*, and DPWG therapeutic recommendations have been made available based on these genotype results to the patient’s healthcare team within one week of starting the drug. It is expected that the primary results of PREPARE will be available in 2021 and, whilst PREPARE is not powered to answer whether individual drug–gene pairs should be implemented, it will address whether or not an overarching pharmacogenomics approach is clinically and/or cost-effective [19].

Recommendations in *CYP2D6* guidelines include: starting with the usual dose of the drug (e.g., in EMs), altering its dose (e.g., in IMs), selecting an alternative drug (e.g., in PM/UMs), or considering increased patient monitoring and therapeutic drug monitoring if available (e.g., in PMs). In a few guidelines (e.g., atomoxetine, tamoxifen), the IM category is further subdivided and specific recommendations are provided according to the presence/absence of the CYP2D6*10 allele (AS 0.25). This metaboliser status classification system has been similarly used in the pharmacogenomic guidelines for other important CYP pharmacogenes (e.g., *CYP2B6, CYP2C9*, *CYP2C19, CYP3A5*), albeit usually without activity scoring because of the lower complexity of genetic variation compared to *CYP2D6.* However, these other CYP pharmacogenes can have different gene-specific considerations, such as an additional rapid metaboliser (RM) category for *CYP2C19*, and no UM group for CYP3A5.

A nationwide study in the Netherlands of over 45 commonly prescribed drugs paired with genotype data found that, overall, the implementation of pharmacogenomics using DPWG guidelines could lead to 5.4% of prescriptions being altered (e.g., dose adjustments or selection of alternative drugs) [94]. For drugs linked to *CYP2D6*, the proportion of patients with an actionable recommendation varied by drug according to which metaboliser statuses were deemed actionable. Thus, for example, only 5% of aripiprazole prescriptions were associated with a recommendation (reduced maximum dose) because only the PM category is considered actionable in DPWG guidance for aripiprazole. However, DPWG guidance recommends optional (for IM or PM patients) or definite (for UM patients) prescription alterations for 47% of tramadol prescriptions. Several other drugs, such as flecainide, venlafaxine and imipramine, were similarly recommended a drug or dosage change based on *CYP2D6* genotype in >45% of cases [94]. The following subsections highlight *CYP2D6* pharmacogenomics in relation to two case-studies: codeine and tamoxifen.

## 8. CYP2D6-Drug Case Studies

### 8.1. Codeine

**Figure 4:**
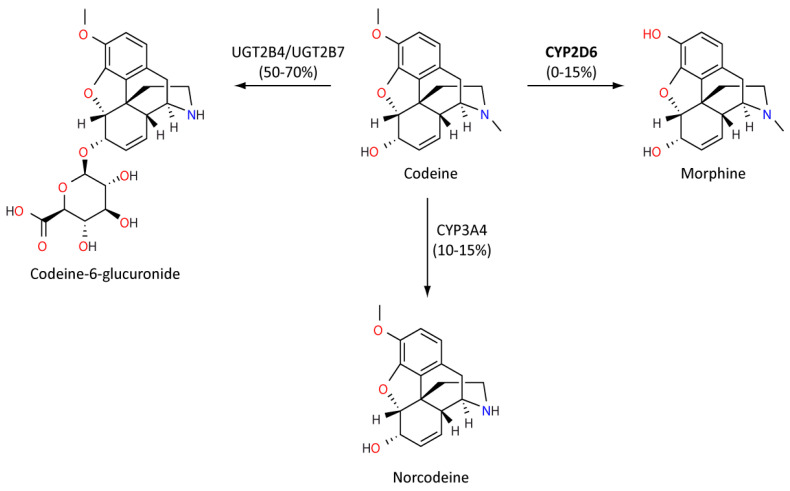
This figure illustrates the CYP2D6-mediated conversion of codeine to morphine. This pathway results in the metabolism of 0–15% of codeine. Other metabolites codeine-6-glucuronide and norcodeine are metabolized by UGT2B4 and UGT2B7, and CYP3A4, respectively, accounting for remaining codeine metabolism. [96]. Secondary metabolites such as morphine-6-glucuronide are not shown for simplicity.

Codeine is an opiate prodrug used primarily as an analgesic agent and to treat diarrhoea and cough. However, codeine can have a range of adverse effects from relatively mild nausea, constipation and headaches, through to drowsiness and serious respiratory depression. Metabolism of codeine into active morphine is dependent on CYP2D6, and so is strongly influenced by *CYP2D6* genotype (Figure 4). Codeine displays a reduced binding potential to mu opioid receptors compared to morphine, resulting in milder analgesic effects. Codeine is also considered to be the safer of the two compounds [95,96]. If an individual is a *CYP2D6* PM, there is an increased likelihood of an insufficient analgesic effect [97]. Conversely, several importantly case reports have identified severe respiratory depression in UM children following tonsillectomies [97,98,99]. Mothers who are *CYP2D6* UMs also appear to have increased morphine present in their breast milk, and this has been associated with severe adverse events in breastfed infants [100].

### 8.2. Tamoxifen

**Figure 5:**
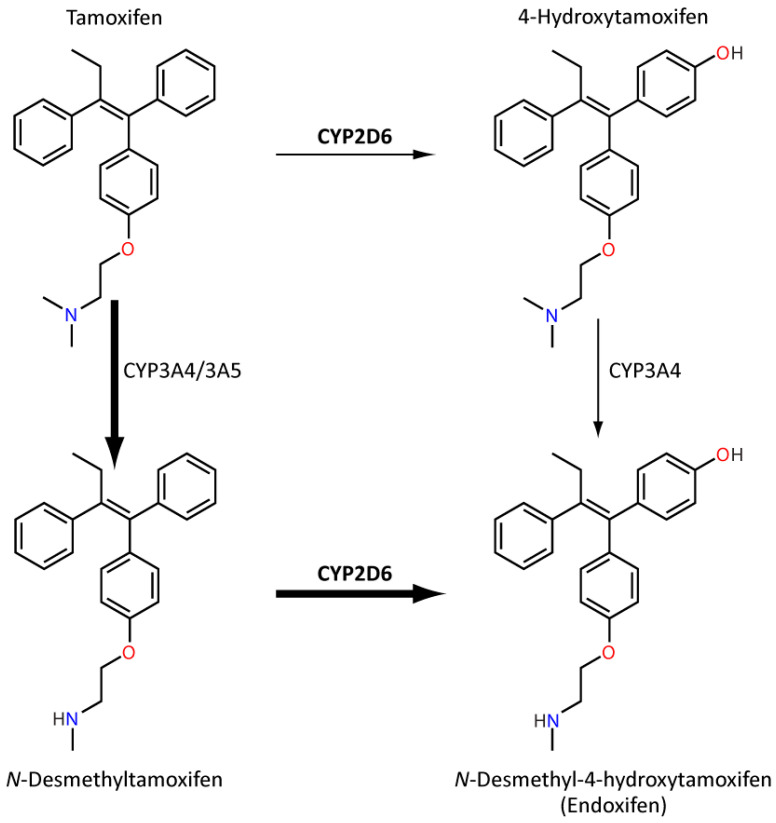
This figure provides a simplified overview focusing on the role of CYP2D6 in the formation of endoxifen. Over 90% of tamoxifen metabolism is CYP3A-mediated N-demethylation to the primary metabolite, N-desmethyltamoxifen, which can be oxidized further by CYP2D6 to endoxifen. Approximately 7% of tamoxifen metabolism is CYP2D6-mediated hydroxylation to 4-hydroxytamoxifen. There is high interindividual variation in endoxifen exposure, which is a significantly more potent anti-oestrogen compared to parent tamoxifen [101,109].

Tamoxifen is a selective oestrogen receptor modulator (SERM) frequently used to reduce the incidence of breast cancer recurrence in pre- and perimenopausal women with oestrogen receptor-positive breast cancer. Although generally well tolerated, it can cause hot flushes, fatigue and nausea amongst other adverse effects. Tamoxifen metabolism is extensive and complex (Figure 5). Nevertheless, an important secondary metabolite, endoxifen, has almost 100-fold greater anti-oestrogenic potency than parent tamoxifen [101]. The major route of endoxifen formation is demethylation via CYP3A4/5 followed by oxidation by CYP2D6 [101]. *CYP2D6* IM/PMs have been associated with reduced endoxifen plasma concentration [102], although the evidence relating *CYP2D6* to clinical outcomes indicative of reduced tamoxifen efficacy, such as breast cancer recurrence and mortality, are inconsistent [101]. Whilst an association has been shown in some studies [103,104], two large studies dispute an effect [104,105]. Notably, tumour tissue was used for genotyping in both these studies which, as mentioned previously, is a major limitation and their findings may not match the genotype of somatic tissue for the same patients. Furthermore, a recent review suggested that a link between *CYP2D6* genotype and tamoxifen adherence was uncovered in these large studies [106] and other studies linked a specific *CYP2D6* haplotype to outcome in tamoxifen-treated patients [107]. Accordingly, clinical guidance for tamoxifen-*CYP2D6* has been developed by CPIC and DPWG as well as other societies, which generally recommends alternative treatment, such as an aromatase inhibitor, in *CYP2D6* IMs and PMs [80,108].

Other common *CYP2D6*-mediated drugs such as amitriptyline have also been found to have strong links between CYP2D6 status and increased risk of ADRs [110]. Overall, the impact of *CYP2D6* genotypes will vary across medical specialities depending on the prevalence of drug prescriptions, which metaboliser phenotypes are associated with altered response for a given drug, and the frequency and severity of a drug’s adverse events.

## 9. Novel Approaches to *CYP2D6* Phenotyping

Many *CYP2D6* variants, including rare variants and complex combinations of haplotypes, have been identified but not assigned a metabolic function. One in vitro state-of-the-art approach to comprehensively determine the empirical functionality of missense variants is saturation mutagenesis with massively parallel functional assays. In this approach, a mutagenesis library of missense variants is constructed that represent all possible amino acid substitutions within a protein and their in vitro functionality is determined. This high-throughput functional screening approach has been successfully applied to *NUDT15* and could be conceivably applied to *CYP2D6* [111]. Analysis of large datasets is another approach to assign functional effects to these undetermined haplotypes, and is being increasingly carried out to interrogate *CYP2D6* [112,113]. One example is the use of sequence data and phenotypic data from >450,000 UK biobank participants [112]. Analysis of these data has been used to elucidate haplotypes, but it was unable to capture structural variations. Technologies such as Nanopore utilise long read sequencing, which is able to sequence, identify and capture structural variants in *CYP2D6* [114].

In recent years, numerous promising approaches have been employed to predict CYP2D6 function, utilising machine learning and deep learning. One interesting example leveraged the observation that CYP2D6 is amongst the most highly expressed CYPs in the brain and so used machine learning to identify imaging patterns within brain perfusion images from functional magnetic resonance imaging (fMRI) associated with CYP2D6 metaboliser groups. This technique was able to detect UM individuals with sensitivity and specificity values of 85–87%, and PM individuals with sensitivity and specificity values of 71–79% [115]. Another recent study utilised a deep learning approach to predict the function of *CYP2D6* genotypes, including those with rare variants, and then derive a subsequent classification model that predicts whether a CYP2D6 haplotype has a normal or reduced function, and was shown to have a high degree of accuracy [116]. Lastly, a neural network model was recently developed and trained on long-read *CYP2D6* sequencing data in conjunction with information on the rate of CYP2D6-mediated tamoxifen metabolism from 561 patients with breast cancer. This model linked *CYP2D6* variation to the tamoxifen metabolic ratio on a continuous scale, rather than categorising it into the conventional discrete metaboliser phenotype groups. Importantly, it could account for 79% of observed interindividual variation in CYP2D6 activity, compared to 54% with the conventional approach. Furthermore, this model could assign enzyme activity to haplotypes containing uncharacterised combinations of variants, and was validated in independent cohorts. Thus, this approach highlights the potential of combining fully phased gene sequence data with machine learning approaches and continuous scale readout to improve pharmacogenotype–phenotype translation [117].

## 10. Veterinary Pharmacogenomics and *CYP2D6* Orthologues

Beyond human pharmacogenomics, interindividual variability in drug response in veterinary practice is also being increasingly explored. However, the scale and implementation of veterinary pharmacogenomics remains limited to date. The primary example of veterinary pharmacogenomics is the identification of an *ABCB1* (also known as *MDR1* and encodes P-glycoprotein) allele that reduces the function of P-gp and has been associated with severe ADRs following administration of the anti-parasite drug, ivermectin, in homozygous individuals [118,119]. This variant is very common in specific canine breeds (e.g., collies and English shepherd dogs) due to selective breeding to historically desired traits, and highlights the requirement for veterinary pharmacogenomic research to go beyond broad species genetics and to target genetics at breed [118,120] and eventually individual level.

To date, the role of *CYP2D6* orthologues in veterinary pharmacogenomics has been poorly elucidated. However, a few examples of *CYP2D6* orthologue genotypes influencing drug metabolism have been studied. Canine and equine orthologues of human *CYP2D6* are *CYP2D15* and *CYP2D50*, respectively [118,121,122]. Four haplotypes have been identified in canine *CYP2D15* (*1, *2, *V1* and *WT2*), all of which have been shown to metabolise celecoxib at different rates in beagles and, additionally, the rate of metabolism of bufuralol was shown to be reduced in the WT2 haplotype [121]. CYP2D15 also has a role in the metabolism of desipramine, dextromethorphan and imipramine [123]. Moreover, the equine orthologue, *CYP2D50*, has been found to harbour SNPs and deletions associated with varying rates of tramadol metabolism, allowing the categorisation of horses into *CYP2D50* PMs and EMs, whereas one horse displayed UM status [122].

## 11. Conclusions

*CYP2D6* is a very important pharmacogene with dozens of common haplotypes. These haplotypes can significantly influence the metabolism and, thus, systemic exposure of *CYP2D6* substrates, leading to variable drug response. As a result, clinical pharmacogenomic guidelines have been developed for at least 48 drugs, which collectively span a wide range of therapeutic indications. Moreover, as approximately 20% of commonly used drugs are metabolised by *CYP2D6*, the ramifications of variable CYP2D6 activity on drug response is likely to be notable in clinical practice. Thus, incorporating *CYP2D6* metaboliser status into routine care is expected to reduce ADRs, save healthcare system resources and improve the efficiency of healthcare sectors. However, there is not yet consensus on which *CYP2D6* variants should be routinely tested for clinical use. Moreover, a fast turnaround, cost-effective, non-labour intensive, accurate and reliable method for analysing *CYP2D6* with the potential for rapid upscaling is required for effective implementation of *CYP2D6* pharmacogenomics in the clinic. However, *CYP2D6* presents a number of technical challenges to mass testing in a healthcare setting. In particular, structural variation in *CYP2D6* can be challenging to identify and characterise, complicates the design of *CYP2D6*-specific amplification and detection methods, and can even mask potentially actionable diplotypes. Nevertheless, several technologies and solutions exist that can identify *CYP2D6* structural variants, although they are, on the whole, relatively expensive, labour-intensive and/or time-consuming for mass testing. The continued efforts to develop new pharmacogenomics guidelines for different drugs will further aid adoption of *CYP2D6* testing in healthcare settings. Finally, novel machine learning approaches that can comprehensively parse *CYP2D6* genotype-phenotype relationships hold significant promise for application to whole genome sequencing initiatives. However, there remains much to do to translate current understanding of *CYP2D6* into tangible benefit for patients in the clinic.

## Figures and Tables

**Table 1:** Available CYP2D6-Drug Clinical Guidelines.

Drug	Drug Class	Guideline Committee Recommendations			PharmGKB Evidence Level
		CPIC	DPWG	Other	
Amiodarone	Antiarrhythmic	-	No Recommendation	-	-
Amitriptyline	Antidepressant	CD or AD	CD or AD	-	1A
Aripiprazole	Antipsychotic	-	CD	-	3
Atenolol	β blocker	-	No Recommendation	-	-
Atomoxetine	ADHD treatment	CD	CD or AD	-	1A
Bisoprolol	β blocker	-	No Recommendation	-	-
Brexpiprazole	Antipsychotic	-	CD	-	-
Carvedilol	β blocker	-	No Recommendation	-	3
Citalopram	Antidepressant	-	No Recommendation	-	3
Clomipramine	Antidepressant	CD or AD	CD or AD	-	1A
Clonidine	Antihypertensive	-	No Recommendation	-	-
Clozapine	Antipsychotic	-	No Recommendation	-	-
Codeine	Analgesic	AD	CD or AD	CPNDs: AD	1A
Desipramine	Antidepressant	CD or AD	-	-	1A
Disopyramide	Antiarrhythmic	-	No Recommendation	-	-
Doxepin	Antidepressant	CD or AD	CD or AD	-	1A
Duloxetine	Antidepressant	-	No Recommendation	-	-
Eliglustat	Gaucher’s disease	-	CD with CM or AD	-	-
Escitalopram	Antidepressant	-	No Recommendation	-	3
Flecainide	Antiarrhythmic	-	CD	-	2A
Fluoxetine	Antidepressant	-	No Recommendation	-	3
Flupenthixol	Antipsychotic	-	No Recommendation	-	-
Fluphenazine	Antipsychotic	-	No Recommendation	-	-
Fluvoxamine	Antidepressant	CD or AD	No Recommendation	-	1A
Gefitinib	Cancer treatment	-	No Recommendation	-	3
Haloperidol	Antipsychotic	-	CD or AD	-	3
Imipramine	Antidepressant	CD or AD	CD	-	1A
Methylphenidate	ADHD treatment	-	No Recommendation	-	4
Metoprolol	β blocker	-	CD or AD	-	2A
Mirtazapine	Antidepressant	-	No Recommendation	-	3
Nortriptyline	Antidepressant	CD or AD	CD or AD	-	1A
Olanzapine	Antipsychotic	-	No Recommendation	-	3
Ondansetron	Antiemetic	AD	-	-	1A
Oxycodone	Analgesic	-	No Recommendation	-	2A
Paroxetine	Antidepressant	CD or AD	AD	-	1A
Pimozide	Antipsychotic	-	CD	-	4
Propafenone	Antiarrhythmic	-	CD or AD	-	2A
Quetiapine	Antipsychotic	-	No Recommendation	-	4
Quinidine	Antiarrhythmic	-	No Recommendation	-	-
Risperidone	Antipsychotic	-	CD or AD	-	1B
Sertraline	Antidepressant	-	No Recommendation	-	3
Sotalol	Antiarrhythmic	-	No Recommendation	-	-
Tamoxifen	Cancer treatment	CD or AD	CD or AD	CPNDS: AD, RNPGx: No Recommendation	1A
Tramadol	Analgesic	-	CD or AD	-	1B
Trimipramine	Antidepressant	CD or AD	-	-	1A
Tropisetron	Antiemetic	AD	-	-	1A
Venlafaxine	Antidepressant	-	CD or AD	-	2A
Zuclopenthixol	Antipsychotic	-	CD or AD	-	3

Data extracted from PharmGKB Clinical Guideline Annotations; guidance is currently available for 48 specific drugs [23]. CPIC: Clinical Pharmacogenetics Implementation Consortium. DPWG: Dutch Pharmacogenetics Working Group. CPNDS: Canadian Pharmacogenomics Network for Drug Safety. RNPGx: French National Network of Pharmacogenetics. CD: Change Dosage based on metabolism status. AD: Alternative Drug recommended based on metabolism status. CM: Co-medication. ADHD: attention deficit hyperactivity disorder. It should be noted that Pharmgkb describes evidence between drug and variants and in some cases different variants within a gene are assigned differing levels of evidence. Although guidelines have been generated, the level of evidence can vary. Thus, PharmGKB assigns *CYP2D6* variant-drug annotations a level based on the strength of evidence. Class 1A and 1B annotations indicate strong evidence based on multiple studies and/or is based on the variant–drug combination having been integrated into pharmacogenetic guidelines or major healthcare system. Class 2A and 2B indicated moderate evidence with an effect seen in multiple studies. Class 3 indicates low evidence, typically a single, highly significant finding of association. Class 4 indicates only preliminary evidence of association.

**Table 2:** Selection of common *CYP2D6* variant frequencies in major population groups.

*CYP2D6* Allele	Frequency				Predicted Function
	AFR	EUR	EAS	SAS	
*xN	0.07	0.03	0.02	0.02	Allele Specific
*1xN	0.03	0.01	0.01	0.01	Increased Metaboliser
*2	0.27	0.34	0.14	0.30	Normal Metaboliser
*3	0.00	0.04	0.00	0.00	Decreased Metaboliser
*4	0.12	0.15	0.00	0.09	None Functional
*4xN	0.02	0.00	0.00	0.00	None Functional
*5	0.04	0.03	0.07	0.03	None Functional
*6	0.00	0.02	0.00	0.00	None Functional
*9	0.00	0.02	0.00	0.01	Decreased Metaboliser
*10/114(A)	0.03	0.00	0.59	0.20	Decreased Metaboliser
*17	0.20	0.00	0.00	0.00	Decreased Metaboliser
*29	0.09	0.00	0.00	0.00	Decreased Metaboliser
*33	0.00	0.01	0.00	0.00	Normal Metaboliser
*35	0.00	0.06	0.00	0.00	Normal Metaboliser
*36	0.00	0.00	0.01	0.01	Decreased Metaboliser
*41	0.03	0.03	0.03	0.08	Decreased Metaboliser

AFR: African Population, EUR: European Population, EAS: East Asian population, SAS: South Asian population. Frequencies for AFR, EUR and EAS populations derived from [63] and SAS from [61]. Predicted function was extracted via the Clinical Pharmacogenetics Implementation Consortium (CPIC) tricyclic antidepressants guideline [64].
